# The double-edged sword effect of performance pressure on public employees: The mediation role of mission valence

**DOI:** 10.3389/fpsyg.2022.992071

**Published:** 2022-10-19

**Authors:** Zhonghua Sheng, Bonai Fan

**Affiliations:** ^1^School of Public Affairs, Zhejiang University, Hangzhou, China; ^2^Research Center for Chinese Organizational Development and Performance Evaluation, Zhejiang University, Hangzhou, China; ^3^Institute for Public Policy, Zhejiang University, Hangzhou, China; ^4^Academy of Neuroeconomics and Neuromanagement, Ningbo University, Ningbo, China

**Keywords:** performance pressure, mission valence, vigor, dedication, inverted U-shaped relationship

## Abstract

Performance pressure is a unique stressor in the public sector. Prior studies revealed that it could be a challenge that stimulates functional behavior (i.e., vigor and dedication) or a threat that leads to dysfunctional consequences (i.e., exhaustion and depersonalization). But these articles failed to provide an integrated theoretical model to explain both phenomena simultaneously. We introduced the double-edged sword effect (also called the “too-much-of-good-thing” effect) of performance pressure to fill this theoretical gap. Furthermore, the mediation role of mission valence was examined to explore the buffet mechanism toward this nonlinear relationship. We collected 1,464 valid questionnaire data from snowball sampling to test the research model. Our results revealed that: (1) performance pressure had an inverted U-shaped relationship with dedication and mission valence; (2) performance pressure hurt vigor rather than the curvilinear relationship; (3) mission valence can mediate the inverted U-shaped relationship between performance pressure and dedication. These empirical findings give theoretical contributions and practical insights to public personnel management.

## Introduction

Performance pressure is a unique stressor in the workplace that is “the urgency to achieve high levels because performance is tied to substantial consequences” (Mitchell et al., 2018). It entails three interrelated factors: shared outcome accountability, heightened scrutiny, and evaluation of work, and significant outcomes (Gardner, 2012). In the private sector, due to the competition of market mechanisms, organization members must pursue the maximization of cost–benefit. Thus, performance management has become one of the most effective incentive tools. In the public sector, however, the problem is more complex. Public employees do not seek to maximize profits, but the legitimacy of organizations rests on the recognition of their citizens. Whether the public agency can effectively implement public policies and provide social services is also driven by performance pressure. Some research finds the performance of public sector health systems has a positive relationship with the life quality of citizens (Sun and Li, 2020). When current performance is inadequate for achieving the desired goal, a negative affective response is linked to the attitude and associated belief (Eisenberger and Aselage, 2009). Therefore, performance pressure plays a more important role in public organizations.

Previous studies hold two paradoxical views about the effect of performance pressure. Some researchers argue that it could be a threat that leads to dysfunctional behavior. Employees facing high-performance pressure may undermine their efforts by engaging in suboptimal processes such as unethical pro-organizational behavior (Chen and Chen, 2021; Xu et al., 2021; Zheng et al., 2021) and not contributing valuable knowledge to teamwork (Bunderson and Sutcliffe, 2002; Willems et al., 2021; Mai et al., 2022), incivility, and retail shrink (Jensen et al., 2019), lead to employees’ instrumental and expressive ties (Ali et al., 2022). Other scholars believe that performance pressure also can be regarded as a challenge that elicits functional behavior. When people are motivated to achieve high performance, they engage in activities to benefit their objectives. People tend to take effort-directing actions (e.g., task planning, knowledge coordination, and morale-building communications) and be more ethical and creative (Ness and Connelly, 2017; Zhang et al., 2017). It should say that both views reflect a certain aspect but fail to capture the full spectrum of the incentive mechanism of performance pressure. The impact of performance pressure is not a simple linear relationship but may also have an inverted U-shaped curvilinear relationship. In other words, in the incentive process of performance pressure, there is a meta-theoretical phenomenon called the “too much of a good thing effect (TMGT)” (Pierce and Aguinis, 2013). Challenge assessment positively mediates the relationship between performance stress and work engagement, while threat assessment negatively mediates the relationship between performance stress and work engagement (Schaufeli et al., 2006; Kundi et al., 2021, 2022). Self-regulation is found to be insignificant mediate in the association between social media addiction and strain (Khan et al., 2021; Tariq et al., 2021; Zheng et al., 2021). Learner proactivity reaps social networks through online interaction, strengthening learner social capital in virtual learning (Khan et al., 2022). Previous studies about the impact of performance pressure on work vigor and dedication are isolated as well as fragmented, and lack theoretical dialog between each other. To be more precise, the appropriate intensity range positively affects the work vigor and dedication. After crossing the inflection point, the marginal incentive effect may decrease, leading to negative psychology such as burnout and exhaustion. To fill the theoretical gap, we further construct the inverted U-shaped mechanism of performance pressure and examines its nonlinear incentive mechanism on it.

In addition, prior studies mostly discussed the influence of performance pressure on individual psychology or behavior (following the “stimulate-response” paradigm or S-R). Still, they less argued the influence of organism psychological resources on organizational behavior. According to Woodworth (1918)‘s dynamic psychology theory, individual psychological states follow the rule of “stimulate-organism-response” (S-O-R). The stimulation from the external environment will be processed by the internal organism of the individual and eventually produce different reactions, such as self-determination motivation, autonomy, competence, and relationships (Deci and Ryan, 1985). For the public sector, mission valence constitutes an important internal psychological coping mechanism (Rainey and Steinbauer, 1999; Wright, 2007; Wright and Pandey, 2011; Wright et al., 2012). The sense of mission valence fuels employees to keep active in work and be willing to participate in completing tasks. They value the mission of the public agency by manifesting the social contributions of the organizational results, which enhances the degree to which people perceive the salience of the organization’s goals (Pasha et al., 2017; Bosak et al., 2021). Therefore, analyzing the mediating effect of mission valence between pressure and work vigor and dedication is urgent.

To sum up, we proposed two research questions (RQs): What are the mechanisms between performance pressure and work vigor as well as dedication among public sector workers? What role does mediation variable mission valence play in this process? By collecting 1,464 valid questionnaire data from snowball sampling, we demonstrated that: (1) performance pressure had an inverted U-shaped relationship with dedication and mission valence; (2) performance pressure hurt vigor rather than the curvilinear relationship; (3) mission valence can mediate the inverted U-shaped relationship between performance pressure and dedication. Our research makes theoretical contributions to public personnel management as well as performance management.

## Theoretical background and hypotheses development

Pressure entails a sense of urgency imposed on someone and is often used as a motivating factor. Performance pressure - a unique source of employee work stress - is a tension to raise performance to attain desirable consequences and avoid negative consequences (Mitchell et al., 2019). It’s based on the belief that meeting or exceeding performance expectations will tie to significant consequences (e.g., promotions, raises, rewards, bonuses, etc.). Failing to meet expectations can result in harmful outcomes (e.g., probation, termination, sanctions, demoted or terminated, etc.). It’s a unique stressor in the workplace because of two reasons: (1) it emphasizes the pursuit of excellence, distinct from time pressure, work overload, or task overburden; (2) it can be anticipated and planned rather than do it at random (Gardner, 2012; Mitchell et al., 2019).

Prior research demonstrated that performance pressure elicits both functional and dysfunctional behavior. Some scholars have shown that it incentives active and positive behavior. Industrial-organizational psychologists find that competition, rewards, and punishments in the workplace can produce the perception that it’s important to meet goals and achieve high performance (Prouska et al., 2016), also referred to as performance pressure. Paradoxically, other research has demonstrated an irony of performance pressure: it impairs individual behavior and boosts dysfunctional behavior. Even though employees are motivated to perform well on the project, a team with high-performance pressure is more likely to involve in performance-detracting behavior (Gardner, 2012). Complex tasks under heightened scrutiny demotivate employees to perform more poorly because of diminished cognitive functioning (Ellis, 2006); they may not focus on collective performance and disrupt team routines. Increased performance pressure involves outcome accountability, which leads people to opt for less-risky approaches that they can easily defend and justify. Employees under high-performance pressure tend to be more cautious and conventional; they may be involved in more heuristic information processing as they rely on socially acceptable knowledge that comes to mind quickly. Moreover, several scholars found that performance pressure is a double-edged sword. Gardner (2012) revealed that performance pressure could enhance team motivation but undermine the use of team knowledge. These inconsistent findings suggest that performance pressure may have an inverted-U shape relationship, resulting in bright and dark side effects for organizations.

### Performance pressure and vigor/dedication

Work engagement is “a positive, fulfilling, affective, motivational state of work-related well-being characterized by vigor, dedication, and absorption” (Schaufeli et al., 2006; Bakker et al., 2008). Among them, vigor and dedication are the two core symptoms of engagement. Vigor refers to high levels of energy and mental resilience, willingness to devote one’s effort to their work, and persistence in the face of difficulties; dedication is characterized by being strongly involved in one’s job and experiencing a sense of inspiration, significance, enthusiasm, and pride (Bakker et al., 2008). Previous studies have shown that work and organizational level antecedents affect vigor and dedication, including demanding citizens (Eldor, 2018), hindrance stressors (Bao and Zhong, 2019), organizational climate (Pecino et al., 2019), empathy, affect, and personality (Martos Martinez et al., 2021), job demands, and resources (Bakker et al., 2008, 2014; Maslach and Leiter, 2008), and daily task performance (Hetland et al., 2022). As for public sector employees, one of the most important factors is performance pressure. It is influenced by bottom-line mentality, which leads to workplace cheating behavior (Kamran et al., 2022). The strength of performance pressure depends on the coping process; during this process, individuals may categorize two types of stressors: challenge and hindrance stressors. The former could yield positive consequences for individuals, creating an innate drive to pursue potential opportunities and advancement, while the latter refers to stressors that damage positive outcomes (Cavanaugh et al., 1998, 2000). Moderate pressure constitutes a high-performance system and stimulates the ability, motivation, and attitude of employees (Takeuchi et al., 2009; Kehoe and Wright, 2013; Xu et al., 2020; Kloutsiniotis et al., 2021); while the pressure from a high-performance system has negative effects, which lead to cheating through anger and self-serving cognitions (Kroon et al., 2009; Spoelma, 2021). According to the additive combinations of latent mechanisms proposed by Haans et al. (2016), the double-edged sword effect of performance pressure leads to an inverted U-shaped. It produces a bright and a dark side (Haans et al., 2016). Thus, we divided the influence of pressure into two categories: “low→medium” and “medium→high”.

In the low→medium intensity, the higher the performance pressure, the more vigor and dedicated the public sector employees would be. Performance is an important indicator to measure the ability of public servants, and appropriate supervision and appraisal are conducive to directing employees’ attention toward implementing policies. Territorial accountability increases the attention of public servants to the work and forces them to prioritize the realization of the target. A high-performance work system facilitates emotional commitment (Takeuchi et al., 2009; Kehoe and Wright, 2013; Xu et al., 2020), enhances organizational citizenship behavior (Kehoe and Wright, 2013; Kloutsiniotis and Mihail, 2020), improves job engagement (Kloutsiniotis and Mihail, 2020). Appropriate pressure is seen as a challenge, which creates an internal focus on potential opportunities and development. This experienced pressure affects public service employees’ well-being, stimulating psychological, emotional, and physiological reactions. Thus, moderate performance pressure can motivate employees to work hard.

In the medium→high intensity, the higher the performance pressure, the less vigor, and dedication the public sector employees would be. Employees would become focused on and overwhelmed by the foreboding and worrisome aspects of the situation (Folkman and Lazarus, 1985). They tend to concentrate on the difficulties of raising performance and the negative consequences that will likely result if performance demands are not met. According to the theory of cognitive stress appraisal (Lazarus and Folkman, 1984), any form of negative stewing is a taxing experience. Threat performance appraisal in motivated situations limits the cardiovascular (i.e., the physical function of an individual’s heart and blood flow) and decreases energy (Seery, 2011; Liu et al., 2022). Moreover, threat appraisals have been found to diminish psychological states, impair concentration, and heighten perceptions that tasks are overly difficult (Hagger et al., 2010). Threat appraisals stemming from performance pressure will deplete self-resources associated with self-regulation. Employees would focus on the impending doom of performance pressure (i.e., threat appraisal) and drain their resources, leaving them in a state of self-regulation.

*Hypothesis 1a: There is an inverted U-shaped relationship between performance pressures and vigor.*

*Hypothesis 1b: There is an inverted U-shaped relationship between performance pressures and dedication.*

### Performance pressure and mission valence

Mission valence was first adopted by Rainey and Steinbauer (1999), who defined it as “the attractiveness of the mission, such as difficult but feasible, reasonably clear and understandable, worthwhile, interesting or exciting, important or influential, and distinctive.” As Barnard (1938) once said, an organization’s ability to use its mission to “satisfy personal ideals relating to nonmaterial, future, or altruistic relations” is one of the most powerful and neglected ways to induce cooperation. In the public sector, mission valence can be viewed as an intrinsic reward that a civil servant’s perceptions of the salience of a public agent’s purpose or social contribution (Rainey and Steinbauer, 1999; Wright, 2007; Wright et al., 2012). Task performance pressure was negatively associated with authentic leadership and psychological capital (Jang, 2022), increasing the perception of co-workers’ undermining (Jang and Kim, 2021). According to the previous literature review, performance pressure may have an inverted U-shaped mechanism on mission valence.

In the low→medium intensity, appropriate performance pressure can enhance mission valence. Performance management is an effective tool for leaders to use to promote agency objectives through target setting and motivating employees to achieve them. Due to its incentive and learning-induced capabilities, performance management has been regarded as a ladder to direct the mission valence of public servants (Pasha et al., 2017). An organization attempts to convey what it values most to public servants by assigning and measuring targets. It enables public employees to focus their efforts and actions toward achieving their goals; then increase their job involvement, incentivizing them to work harder. Moderate pressure from performance appraisal helps civil servants establish a sense of mission and value (Guerrero and Chenevert, 2021). Performance pressure reflects the importance of the task at work. Organization members can stimulate the recognition of their tasks, enhancing the sense of mission valence. Therefore, moderate performance pressure with clear indicators helps public servants relate their tasks to the broader organizational mission and understand how a performance improvement may affect the organization and society (Pasha et al., 2017).

In the medium→high intensity, too much performance pressure leads to a decrease in mission valence. Extrinsic rewards negatively affect intrinsic motivation (Eisenberger and Aselage, 2009). According to self-determination theory (Deci and Ryan, 1985), internal motivation is the key factor affecting behavior, whereas external motivation sometimes has an erosive effect on self-determination motivation. Extrinsic rewards can drive out intrinsic motivation, particularly when the employees are intrinsically motivated (Frey, 1997; Ryan and Deci, 2000; Canton, 2005; Georgellis et al., 2011). Higher extrinsic rewards or high-powered incentives reduce the propensity of intrinsically motivated employees (Georgellis et al., 2011). Performance-oriented human resource system could improve employees’ willingness for self-development, but it may also reduce their moral awareness, which increases unethical behavior (Zhang et al., 2021).

*Hypothesis 2: There is an inverted U-shaped relationship between performance pressures and mission valence.*

### The mediating role of mission valence

The external performance pressure may have an inverted U-shaped relationship with vigor and dedication. The internal mission valence plays a buffer role in this mechanism. The sense of mission valence may expand the positive effect of performance pressure and alleviate the incentive distortion.

On the one hand, mission valence helps enhance the positive effect of performance pressure on vigor and dedication. Because the subjective experience of performance pressure is internalized, mission valence can differ. Appropriate performance management is conducive to guiding people to establish a correct mission, stimulates their sense of responsibility and inspiration, enhances spiritual vitality, and fuels vigor. At the same time, establishing a shared vision and mission is conducive to stimulating team members’ internal drive and promoting cooperation among organization members. Transformational leaders can help alleviate burnout among hospital staff who operate in a challenging and stressful environment (e.g., performance pressure) by increasing the perceived attractiveness or salience of an organization’s mission (Bosak et al., 2021; Guerrero and Chenevert, 2021). And mission valence orients employees to regard their works as part of the organization’s general purpose, accentuating the responsibility of public servants toward the organization and society (Ahn, 2022). It encourages civil servants to transcend their interests in pursuing public goods. Performance pressure involves heightened evaluation and consequences, which increase arousal, physical and mental efforts, and greater persistence in facing difficulties. Therefore, in this circumstance, employees should take action to focus, engage, and prolong their efforts.

On the other hand, mission valence helps buffer the corrosive effect of performance pressure on vigor and dedication. Performance pressure promotes organization members to establish common values and are willing to treat external pressure with positive psychological cognition, change their cognition of stress, promote career involvement, and work engagement, and improve individual mental health and dedication. Facing the external pressure brought by the performance appraisal system, the mission valence changes its internal cognition, provides a sense of task significance, holds a positive meaning (Guerrero and Chenevert, 2021), improves its resilience, and brings dedication and investment.

*Hypothesis 3a: There is a positive indirect effect in the inverted U-shaped relationship between performance pressure and vigor through mission valence.*

*Hypothesis 3b: There is a positive indirect effect in the inverted U-shaped relationship between performance pressure and dedication through mission valence.*

**Figure 1:**
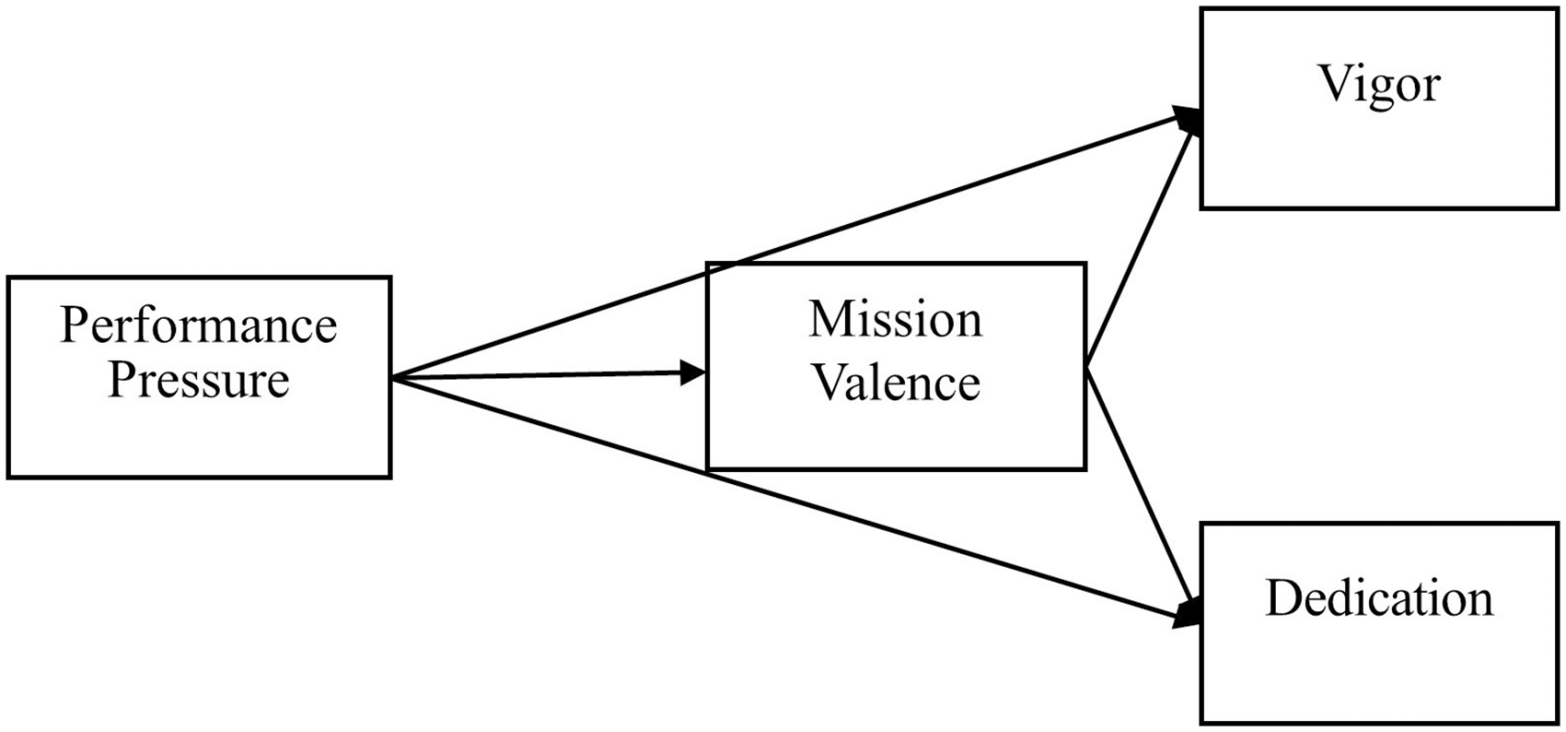
Research model.

The theoretical model is depicted in Figure 1.

## Measurement methods

We adopted the questionnaire survey method to test the theoretical model. Firstly, we defined the connotation and denotation of all variables and selected validated scales to guarantee the quality of the measures. Secondly, this study used convenience sampling to investigate the grassroots public employees in eastern, western, and central regions of China to ensure sample representativeness. Finally, we tested all variables’ reliability, validity, and common method bias to ensure high psychometrical properties.

### Participants

The target population of our study was grassroots public employees in the Chinese public sector. 1,464 public employees were involved in this research: 581 (39.7%) were female, the average rank was 3.73 (*SD* = 1.702), the average education was 4.94 (*SD* = 0.601), the average tenure was 4.35 (*SD* = 1.552), and the average work hour was 3.92 (*SD* = 1.041). The studies involving human participants were reviewed and approved by our university. The patients/participants provided their written informed consent to participate in this study. As a criterion for inclusion, we evaluated public sector employees who were working at the grassroots level or had extensive grassroots work experience.

### Instruments

Since all measurement items were adopted from English scales, we conducted the back-translation procedure of Brislin (1970) to better adapt to the Chinese context. Three Ph.D. students were asked to translate the English items into Chinese; another student translated them back into English. The translation was valid if the back translation was consistent with the original one. After that, several rounds of expert consultation meetings were held to invite experts in public management, psychology, political science, and other fields to evaluate the questionnaire in a back-to-back manner and put forward suggestions for revision independently. At the same time, we conducted several field investigations and semi-structured interviews with front-line public servants, practitioners, and leaders and then revised certain items based on their feedback. After that, a small-scale pretest was conducted for this questionnaire. Combined with quantitative analysis and participants’ comments, we rephrased certain sentences and eliminated ambiguous items to improve the clarity of our scales, thus forming the final questionnaire. Unless indicated, participants responded to items based on a 7-point Likert scale of 1 = strongly disagree, 7 = strongly agree. The complete list of all items is presented in Appendix A.

#### Performance pressure

Perceived performance pressure was measured with four items from Mitchell et al. (2018). Sample items include “Today, I feel that if I do not produce at high levels, my job will be at risk” and “Today, I feel tremendous pressure to produce results.” The Cronbach’s alpha was 0.848.

#### Vigor

Vigor was assessed with six items from the Utrecht Work Engagement Scale (UWES) in Schaufeli et al. (2006). Sample items include “At my work, I feel bursting with energy” and “At my job, I feel strong and vigorous.” The Cronbach’s alpha was 0.925.

#### Dedication

Dedication was also measured with five items from Schaufeli et al. (2006). Example items are: “I am enthusiastic about my job,” and “My job inspires me.” The five items showed good internal consistency with Cronbach’s alpha of 0.872.

#### Mission valence

Mission Valence was measured with four items from Wright and Pandey (2011). Example items are: “For me, the mission of this organization is exciting,” and “This organization provides valuable public services.” The Cronbach’s alpha was 0.874.

#### Control variables

Many previous studies have confirmed that gender, age, education, marriage, and other demographic characteristics affect vigor, dedication, and burnout (Cordes and Dougherty, 1993; Maslach et al., 2001). Existing literature about Chinese public employees regarded rank, age, education, tenure, and so on as the controlled variables (Bao and Zhong, 2019; Lu and Guy, 2019; Xie and Yang, 2021). We controlled the gender, age, rank, workhour, tenure, education, and job category. Among them, the classification of job categories refers to the relevant provisions of the Civil Servant Law of China and was divided into administrative law enforcement (JC_ale), professional technology (JC_pt), comprehensive administration (JC_ca), and others, thus generating three dummy variables. Variable descriptions are demonstrated in Table 1.

**Table 1:** Description of variables.

Categorical	Variables	Definition and source
Independent variable	Performance pressure	Mitchell et al. (2018); Mitchell et al. (2019)
Mediation variable	Mission valence	Wright and Pandey (2011); Wright et al. (2012)
Dependent variables	Vigor	Schaufeli et al. (2006)
	Dedication	Schaufeli et al. (2006)
Control variables	Gender	1 = male, 2 = female
	Age	1 = 30 years old and below, 2 = 31–35 year old, 3 = 36–40 year old, 4 = 41–45 year old, 5 = 46–50 year old, 6 = 51–55 year old, 7 = 56–60 year old
	Rank	1 = none, 2 = division personnel and clerks (ke-yuan), 3 = deputy division directors and deputies at the sub-division level (fu-ke-ji), 4 = division directors and chiefs at the sub-division level (zheng-ke-ji), 5 = deputies at the section level (fu-chu-ji), 6 = division directors and chiefs at the section level (zheng-chu-ji).
	Education	1 = junior high school degree, 2 = high-school degree (including technical secondary school education), 3 = bachelor’s degree (including associate degree), 4 = master’s degree，5 = doctoral degree, 6 = others.
	Position	1 = administrative law enforcement, 2 = professional technology，3 = comprehensive administration, 4 = others.
	Tenure	1 = work in grassroots for less than 1 year, 2 = 1–3 years, 3 = 4–5 years, 4 = 6–8 years, 5 = 9–11 years, 6 = 12–15 years, 7 = 16 years above.
	Workhour	1 = The average workhour is less than 4 h per day, 2 = 4–6 h per day, 3 = 6–8 h per day, 4 = 8–10 h per day, 5 = 10–12 h per day, 6 = 12–14 h per day, 7 = 14 h above per day.

### Procedure

Considering the difficulties in surveying Chinese public sector employees, we recruited survey subjects by the convenience sampling method recommended by Manion (1994) to ensure sample representativeness as far as possible. We adopted field and auxiliary investigation methods in the data collection process. To increase the representativeness of our sample, we first purposefully selected the provinces from the eastern, central, and western regions according to factors such as the degree of economic development, differences in the work characteristic, and endowment of social resources. Then, we choose the counties (districts) based on the state of economic development. With the help of local personnel, we recruited front-line and grass-roots party and government employees from representative departments. The sample size was determined by the type of department and the size of the organization. At the same time, to reflect the overall picture of grassroots public employees and avoid selection bias in sampling, we combined the snowball sampling method to conduct an auxiliary survey on Master of Public Administration (MPA) students in a university to supplement empirical data. Data were collected in 2021 and distributed through electronic and paper questionnaires to ensure anonymity and confidentiality. The approximate time to complete the survey was 10 to 15 min. The academic purpose of the survey was introduced at the beginning of the survey, and anonymity and confidentiality were guaranteed. A total of 1,534 questionnaires were distributed, and 1,486 were finally collected. After excluding invalid questionnaires such as the omission of data, position above the department level (Zhengchu Ji), and subjects filling in the same answer, the final valid number was 1,464, with an effective questionnaire rate of 98.52% (Table 2). Among these 1,464 respondents, we also examined the possibility of the nonresponse bias by using two-tailed T-tests of variables on demographic characteristics (gender, age, rank, and education) and Chi-square tests on the region. The results were all insignificant, which revealed that there is no nonresponse bias.

**Table 2:** Description of the sample.

Category	Value	Frequency (n)	Percentage (%)
Gender	Men	581	39.686
	Women	883	60.314
Location	Eastern region	364	24.863
	Middle region	601	41.052
	Western region	499	34.085
Rank	None	263	17.964
	Division personnel and clerks	267	18.238
	Deputy division directors and deputies at the sub-division level	363	24.795
	Division directors and chiefs at the sub-division level	329	22.473
	Deputies at the section level	139	9.495
	Division directors and chiefs at the section level	103	7.036
Age	30 years old and below	233	15.915
	31–40	465	31.762
	41–50	514	35.109
	More than 50-year-old	252	17.213

## Data analysis

### Measurement model

We used SPSS 23.0 to analyze the reliability of examined variables, Amos 24.0 to test the validity, and Stata 16.0 to test the hypotheses used by hierarchical regression analysis. The reliability and validity analysis was used to test the psychometric properties of our research model. The Cronbach’s α values were all greater than 0.7, which means that all variables had good reliability. In the validity test, we first used exploratory factor analysis (EFA) to test examined variables. We removed one item of vigor and two items of dedication from the original scales because these items did not fit perfectly with EFA. The EFA results show that the KMO value was 0.890, the significance of Bartlett’s sphericity test was *p* < 0.001, the total variance interpretation was 75.903%, the factor loading of each item in its component was greater than 0.5 (Table 3), which proved to have good validity.

**Table 3:** Exploratory factor analysis (EFA) results.

Items	Component			
	Vigor	Dedication	Mission valence	Performance pressure
VI 1	0.895	0.209	−0.074	0.172
VI 2	0.895	0.120	−0.034	0.184
VI 3	0.848	0.102	0.029	0.178
VI 4	0.811	0.327	−0.080	0.141
VI 5	0.707	0.494	−0.087	0.125
DE 1	0.230	0.838	−0.176	0.155
DE 2	0.231	0.836	−0.178	0.103
DE 3	0.110	0.819	−0.148	0.118
DE 4	0.420	0.746	−0.229	0.113
MV 1	−0.108	−0.187	0.881	−0.005
MV 2	−0.059	−0.181	0.878	−0.001
MV 3	−0.045	−0.086	0.815	0.004
MV 4	0.033	−0.129	0.780	−0.039
PP 1	0.149	0.125	−0.028	0.857
PP 2	0.090	0.151	0.040	0.835
PP 3	0.141	0.111	−0.045	0.827
PP 4	0.222	0.033	−0.012	0.727

Extraction method: the principal component analysis. Rotation method: Kaiser normalized maximum variance method. Rotation converges after 5 iterations.

We further adopted confirmatory factor analysis (CFA) to test the discriminant validity in Amos 24.0 and selected nine goodness of fit indexes: Chi-square (*χ*^2^), degree of freedom (*df*), Chi-square/degree of freedom (*χ*^2^/*df*), root mean square error of approximation (RMSEA), goodness-of-fit index (GFI), normed fit index (NFI), incremental fit index (IFI), comparative fit index (CFI), and root mean square residual (RMR). The CFA results were shown in Table 4. The hypothesized four-factor model fitted data well (*χ*^2^ = 387.761, *χ*^2^/*df* = 4.672, RMSEA = 0.056, GFI = 0.956, NFI = 0.966, IFI = 0.73, CFI = 0.973, RMR = 0.092). The fitting index of the four factors model was better than other competitive models. These results indicated that our research has good discriminate validity. We concluded that our theoretical model was distinct and solid.

**Table 4:** Confirmatory factor analysis (CFA) results.

Model	*χ* ^2^	*df*	*χ*^2^/*df*	RMSEA	GFI	NFI	IFI	CFI	RMR
Four-factor model^a^	387.761	83	4.672^***^	0.056	0.956	0.966	0.973	0.973	0.092
Three-factor model^b^	432.911	84	5.154^***^	0.060	0.951	0.962	0.969	0.969	0.139
Two-factor model^c^	437.596	85	5.148^***^	0.060	0.950	0.961	0.969	0.969	0.140
One-factor model^d^	461.813	86	5.370^***^	0.061	0.947	0.959	0.967	0.967	0.152

a Performance pressure; mission valence; vigor; dedication.

b Performance pressure + mission valence; vigor; dedication.

c Performance pressure + mission valence + dedication; vigor.

d Performance pressure + mission valence + vigor + dedication.

***p < 0.001.

### Common method biases

As our study used the self-report method to collect data, common method biases (CMB) might exist. Therefore, it’s necessary to adopt procedural and statistical techniques and remedies (Podsakoff et al., 2003). In procedural control, this study protects respondent anonymity, reduces evaluation apprehension, and counterbalances question order during the investigation. In the statistical control, we applied Harman’s single-factor test. The results showed that a total of four factors were extracted, among which the initial eigenvalue of the first factor was 5.929, and its explained variance was 37.055%, which was lower than the 50% benchmark recommended by Podsakoff et al. (2003). It demonstrated that CMB had no significant effect on the research model so that we could conduct hypothesis testing.

### Correlation analysis

The Pearson correlation analysis (including means, standard deviations, and correlation coefficients) was reported in Table 5. Performance pressure was negatively related to vigor (*r* = −0.376, *p* < 0.01) and dedication (*r* = −0.293, *p* < 0.01), and was not significantly related to mission valence (*r* = −0.053, *p* > 0.05). This insignificant effect shows that there is not a simple linear relationship between performance pressure and mission valence, but there may be a curve relationship. We further test their relationship in the subsequent regression analysis. Mission valence was positively related to vigor (*r* = 0.135, *p* < 0.01), and dedication (*r* = 0.358, p < 0.01). Age, rank, and tenure were positively related to vigor (*r* = 0.081, p < 0.01; *r* = 0.062, *p* < 0.05; *r* = 0.084, *p* < 0.01) and dedication (*r* = 0.085, p < 0.01; *r* = 0.079, p < 0.01; *r* = 0.085, *p* < 0.01). Workhour was negatively related to vigor (*r* = −0.305, p < 0.01) and dedication (*r* = −0.058, *p* < 0.05). Gender was not significantly related to vigor (*r* = 0.025, *p* > 0.05) or dedication (*r* = 0.000, *p* > 0.05). These results are consistent with the theoretical hypothesis and provide the premise for the subsequent regression analysis.

**Table 5:** Means, standard deviations, and correlations among variables.

	Mean	SD	1	2	3	4	5	6	7	8	9	10	11	12	13
1. Gender	1.372	0.484	1												
2. Age	2.542	0.906	−0.215**	1											
3. Rank	3.727	1.702	−0.205**	0.351**	1										
4. Education	4.942	0.601	0.000	−0.202**	0.248**	1									
5. Tenure	4.352	1.552	−0.209**	0.853**	0.378**	−0.182**	1								
6. WorkHour	3.923	1.041	−0.073*	−0.152**	0.025	0.071*	−0.109**	1							
7. JC_ale	0.082	0.274	−0.048	0.009	−0.002	0.003	0.017	−0.102**	1						
8. JC_cm	0.634	0.482	−0.088**	−0.031	0.371**	0.192**	−0.021	0.112**	−0.393**	1					
9. JC_pt	0.144	0.351	0.004	0.034	−0.289**	−0.030	0.016	−0.045	−0.122**	−0.539**	1				
10. PP	5.927	1.048	0.028	0.001	−0.049	−0.028	0.004	0.137**	−0.045	−0.008	0.008	**0.848**			
11. MV	4.699	1.467	−0.035	0.090**	0.010	−0.078**	0.077**	−0.012	0.007	−0.081**	0.068*	−0.053	**0.874**		
12. DE	3.912	1.682	0.000	0.085**	0.079**	−0.016	0.085**	−0.058*	0.049	0.009	−0.012	−0.293**	0.358**	**0.872**	
13. VI	2.688	1.393	0.025	0.081**	0.062*	−0.047	0.084**	−0.305**	0.023	−0.035	0.033	−0.376**	0.135**	0.452**	**0.925**

PP, performance pressure, MV, mission valence, DE, dedication, VI, vigor. *N* = 1,464, Cronbach’s alpha is reported in diagonal.

* *p*< 0.05;

** *p*< 0.01.

The bold values in the diagonal are Cronbach’s alpha.

### Hypothesis testing

Before hypothesis testing, we conducted mean-centering for each variable to reduce multicollinearity problems. Since all constructs were continuous variables, the ordinary least squares (OLS) method was used for regression analysis. We controlled gender, age, rank, workhour, tenure, education, and job categories. To achieve the optimal sample size ratio to the number of parameters, we conducted item parceling on all variables (Little et al., 2002; Williams et al., 2009). Since this study aims to distinguish the differences among core variables rather than explore the relationship between items, it is reasonable to pack variables with many items (Little et al., 2013). We used Stata 16.0 for hierarchical regression analysis.

#### Inverted-U test

The regression equation of the inverted U-shaped mechanism is as follows:


$$Y_{i}=\vartheta +\gamma X_{i}^{2}+\omega X_{i}+\varphi Z_{i}+\varepsilon_{i},i=1,2,3,4\ldots n\;\left(1\right)$$

In the regression equation, Y is the dependent variable of each individual, ϑ is the constant, X is the independent variable, Z is the control variable, and ε is the error. The inflection point *δ* = −ω/2γ. Model 2 in Table 6 reveals that performance pressures are negatively related to vigor (*β* = −0.454, *p* < 0.001). Model 3 reveals that performance pressures are negatively related to vigor (*β* = −0.482, *p* < 0.001) while performance pressure square is not significantly related to vigor (*β* = −0.018, *p* > 0.05). Hypothesis 1a did not get supported. Model 6 reveals that performance pressures are negatively related to dedication (*β* = −0.465, *p* < 0.001). Model 7 reveals that performance pressures are negatively related to dedication (*β* = −0.596, *p* < 0.001), and performance pressure square is negatively related to dedication (*β* = −0.082, *p* < 0.01). The inflection point of performance pressure on dedication is *δ*_1_ = −3.634, which is inside the scope of centralization of performance pressure (x∈ [−4.927, 1.073]), indicating that there is an inverted U-shaped curve. Hypothesis 1b got supported.

**Table 6:** Regression results for the main effect and mediating effect testing.

variables	Vigor					Dedication					Mission Valence		
	Model 1	Model 2	Model 3	Model 4	Model 5	Model 6	Model 7	Model 8	Model 9	Model10	Model11	Model12	Model13
*Gender*	0.0648	0.0980	0.0993	0.0730	0.104	0.104	0.138	0.144	0.132	0.166	−0.0682	−0.0624	0.0648
	(0.0875)	(0.0811)	(0.0812)	(0.0865)	(0.0803)	(0.109)	(0.104)	(0.103)	(0.1000)	(0.0955)	(0.0922)	(0.0922)	(0.0875)
*Age*	−0.0789	−0.0630	−0.0622	−0.0922	−0.0746	0.0617	0.0780	0.0816	0.0165	0.0368	0.110	0.113	−0.0789
	(0.0983)	(0.0950)	(0.0956)	(0.0977)	(0.0939)	(0.106)	(0.102)	(0.104)	(0.104)	(0.0991)	(0.0971)	(0.0983)	(0.0983)
*Rank*	0.0804^*^	0.0637^*^	0.0631^*^	0.0765^*^	0.0606^*^	0.0624	0.0452	0.0427	0.0490	0.0320	0.0325	0.0296	0.0804^*^
	(0.0320)	(0.0300)	(0.0302)	(0.0316)	(0.0296)	(0.0364)	(0.0347)	(0.0348)	(0.0345)	(0.0327)	(0.0325)	(0.0326)	(0.0320)
*Education*	−0.1122	−0.1189	−0.121	−0.0942	−0.103	−0.0658	−0.0727	−0.0800	−0.00456	−0.0196	−0.149	−0.150	−0.112
	(0.0917)	(0.0916)	(0.0919)	(0.0901)	(0.0896)	(0.0883)	(0.0861)	(0.0869)	(0.0887)	(0.0854)	(0.0761)	(0.0769)	(0.0917)
*Tenure*	0.0477	0.0547	0.0533	0.0491	0.0557	0.0312	0.0384	0.0323	0.0359	0.0381	−0.0113	−0.0101	0.0477
	(0.0537)	(0.0509)	(0.0515)	(0.0538)	(0.0504)	(0.0642)	(0.0615)	(0.0625)	(0.0646)	(0.0612)	(0.0593)	(0.0601)	(0.0537)
*Workhour*	−0.404^***^	−0.3371^***^	−0.336^***^	−0.405^***^	−0.339^***^	−0.0716	−0.00318	−0.000552	−0.0754	−0.00943	0.00928	0.0209	−0.404^***^
	(0.0391)	(0.0386)	(0.0385)	(0.0387)	(0.0382)	(0.0517)	(0.0509)	(0.0506)	(0.0472)	(0.0457)	(0.0468)	(0.0478)	(0.0391)
*JC_ale*	−0.0121	−0.0844	−0.0798	0.000444	−0.0724	0.405	0.331	0.352	0.447^*^	0.390^*^	−0.104	−0.116	−0.0121
	(0.174)	(0.167)	(0.166)	(0.171)	(0.165)	(0.211)	(0.210)	(0.205)	(0.196)	(0.191)	(0.199)	(0.199)	(0.174)
*JC_ca*	−0.0112	−0.0363	−0.0329	0.0162	−0.0124	0.150	0.125	0.140	0.244	0.225	−0.227	−0.232	−0.0112
	(0.124)	(0.117)	(0.117)	(0.124)	(0.116)	(0.152)	(0.145)	(0.144)	(0.142)	(0.136)	(0.136)	(0.136)	(0.124)
*JC_pt*	0.178	0.1465	0.155	0.162	0.133	0.161	0.128	0.167	0.105	0.104	0.135	0.130	0.178
	(0.161)	(0.149)	(0.149)	(0.161)	(0.149)	(0.194)	(0.183)	(0.183)	(0.188)	(0.177)	(0.168)	(0.168)	(0.161)
*PP*		−0.4535^***^	−0.482^***^		−0.445^***^		−0.465^***^	−0.596^***^		−0.525^***^		−0.0787	−0.1826^***^
		(0.0389)	(0.0467)		(0.0387)		(0.0469)	(0.0595)		(0.0570)		(0.0450)	(0.0563)
*PP^2^*			−0.0179					−0.0818^**^		−0.0566^*^			−0.0652^**^
			(0.0247)					(0.0280)		(0.0259)			(0.0247)
*MV*				0.121^***^	0.103^***^				0.411^***^	0.387^***^			
				(0.0275)	(0.0257)				(0.0344)	(0.0329)			
*Constant*	4.413^***^	4.157^***^	4.183^***^	4.344^***^	4.102^***^	3.699^***^	3.436^***^	3.553^***^	3.463^***^	3.312^***^	0.575	0.531	4.413^***^
	(0.503)	(0.486)	(0.488)	(0.497)	(0.478)	(0.564)	(0.544)	(0.548)	(0.545)	(0.519)	(0.492)	(0.497)	(0.503)
*N*	1,464	1,464	1,464	1,464	1,464	1,464	1,464	1,464	1,464	1,464	1,464	1,464	1,464
*R* ^2^	0.103	0.216	0.217	0.119	0.228	0.016	0.098	0.107	0.142	0.217	0.019	0.022	0.103
△*R*^2^		0.113	0.114	0.016	0.212		0.082	0.091	0.126	0.114		0.003	0.084
*F*-Value	16.38	32.43	31.21	18.31	33.50	2.15	11.89	13.16	18.84	33.86	2.65	2.56	3.18
VIF	2.04	1.94	2.00	1.94	1.86	2.04	1.94	2.00	1.94	1.92	2.04	1.94	2.00

PP, performance pressure, MV, mission valence.

* *p* < 0.05;

** *p* < 0.01;

*** *p* < 0.001.

Model 10 reveals that performance pressures are not significantly related to mission valence (*β* = −0.079, p > 0.05). Model 11 reveals that performance pressures are negatively related to mission valence (*β* = −0.183, *p* < 0.001), and performance pressure square is negatively related to mission valence (*β* = −0.065, *p* < 0.01). The inflection point of performance pressure on dedication is *δ*_2_ = −1.408, which is inside the scope of centralization of the independent variable (x∈ [−4.927, 1.073]). This indicates an inverted U-shaped curve; thus, Hypothesis 2 was supported.

The VIF of each regression equation is <3; thus, there is no multicollinearity.

#### Mediation effect analysis

**Figure 2:**
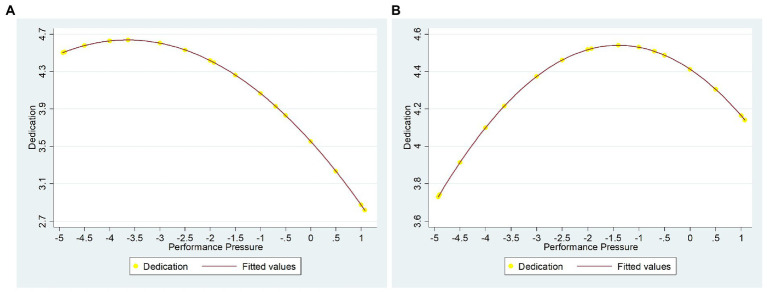
The incentive effect of performance pressure on dedication. **(A)** the direct effect of performance pressure on dedication; **(B)** the moderation effect of mission valence between performance pressure and dedication. The dotted line indicates the midpoint, and the solid line indicates the inflection point.

Based on the mediating effect test proposed by Baron and Kenny (1986), this study adopted the hierarchical regression model to test the mediating effect of mission valence. The analysis results are shown in Models 7, 8, and 12. The statistical results show that: (1) performance pressure square is negatively related to dedication (*β* = −0.082, *p* < 0.01); (2) performance pressures square is negatively related to mission valence (*β* = −0.065, *p* < 0.01); (3) put the performance pressure square and mission valence together into the equation, mission valence is positively related to dedication (*β* = 0.387, *p* < 0.001), and performance pressure square is negatively related to dedication (*β* = −0.057, *p* < 0.05). The path coefficient of the performance pressure square to dedication becomes decreases. It demonstrates that mission valence partially mediates the inverted U-shaped relationship between performance pressure and dedication. After adding the mediation variable of mission valence, the inflection point of performance pressure on dedication is *δ*_3_ = −4.638, which is inside the scope of centralization of independent variable performance pressure (x∈ [−4.927, 1.073]). It indicates that there is an inverted U-shaped curve. Compared with the midpoint of the independent variable (*α* = −1.927), the inflection point is located on the right, indicating that under the mediating effect of mission valence, the incentive effect of performance pressure on dedication is “asymmetric,” and its positive incentive effect expands. Thus, Hypothesis 3b got supported (Figure 2). Besides, we further control the variable of mission valence, and the statistical results in Model 5 show that performance pressure is negatively related to vigor (*β* = −0.445, *p* < 0.001), and the path coefficient of the performance pressure to vigor becomes decreases. Thus, Hypothesis 3a did not get support. The VIF of each regression equation is less than 3; thus, there is no multicollinearity.

To further examine the mediation effect, we follow the advice of Bauer et al. (2006) to explore indirect effects and conduct a bootstrapping analysis. The bootstrap method extracts 5,000 samples from the original data (with substitution) and calculates the mediating effects in each sample. The point estimate of the mediation effect is the mean of these 5,000 samples, with a 95% confidence interval calculation. Therefore, if the confidence intervals do not contain zero, the point estimate is significant at the level indicated (Preacher and Hayes, 2004). Thus, we tested Hypothesis 3a and 3b by conducting an overall indirect model using the PROCESS method (use Models 4) to assess the significance of the mediation effects of our model (Hayes, 2013). Table 7 shows the research results. The bootstrapped point estimate and confidence intervals (CI) results show that the indirect effect of performance pressure on vigor is-0.4454 (*p* > 0.05), and the CI is between-0.0142 and 0.007 (including zero). This means Hypothesis 3a did not get support. While the indirect effect of performance pressure square to dication is-0.0095 (p < 0.05), and the CI is between-0.0043 and-0.003 (not including zero). This means Hypothesis 3b gets supported.

**Table 7:** Bootstrapped point estimate and confidence intervals (CI) of the mediating effects of mission valence on the indirect relationship between performance pressure and dedication/vigor.

Mediation path *via* mission valence	X-M	M-Y	X-Y total effect	X-Y direct effect	The mediation effect		Bootstrapping (95%) CI	
					Effect	SE	Lower limit	Upper limit
Performance pressure → vigor	−0.0787	0.1031^***^	−0.4535^***^	−0.4454	−0.0081	0.0052	−0.0142	0.0007
Performance pressure^2^ → dedication	−0.0095^*^	0.3884^***^	−0.0465^***^	−0.0428^***^	−0.0037	0.0017	−0.0043	−0.0003

All direct, indirect, and total effects include controlling the variables: gender, age, rank, workhour, tenure, education, and job category (including JC_ale, JC_pt, JC_ca). *N* = 1,464; Bootstrap sample size = 5,000. X: performance pressure; M: mission valence; Y: vigor and dedication. CI, confidence interval; SE, standard error.

* *p* < 0.05;

*** *p* < 0.001.

#### Robustness test

According to the suggestion of Haans et al. (2016), we adopted Lind and Mehlum (2010)‘s three-step method to test the robustness of the inverted U-shaped relationship, taking H2b as an example: (1) the regression coefficient of the square term of the independent variable performance pressure was significantly negative (*β* = −0.065, *p* < 0.01); (2) The slope of the minimum point of the independent variable is significantly positive (*β* = 0.461, *p* < 0.05), and the slope of the maximum point is significantly negative (*β* = −0.323, *p* < 0.001); (3) The Filler 95% confidence interval of the inflection point is (−3.690, −0.750), which is within the range of centralized independent variable performance pressure (x∈[−4.927, 1.073]). The above analysis shows that the inverted U-shaped relationship is still valid, and the research conclusion remains robust.

## Discussion

### Theoretical implications

Our research has the following theoretical contributions. First, compared with previous studies, we have a dialog with performance pressure theory, and reveal various effects of performance stress on different dimensions of engagement. The initial contextualization suggests that the incentive effect of performance pressure on individuals is either a positive facilitator (Ness and Connelly, 2017) or a negative hindrance (Chen and Chen, 2021; Willems et al., 2021; Mai et al., 2022). But in fact, these two effects may exist at the same time. That is, moderate performance pressures promote individual vigor and dedication. Performance pressure controlled at an appropriate intensity urges public sector employees to consider it a challenge, stimulating their work vitality, motivation, and attitude, and promoting dedication. However, when the intensity of pressure is too high, employees will regard it as a threat and will face the risk of being accounted for if they continue to dedicate themselves. Therefore, public sector workers who prefer risk-averse will take blame avoidance strategies to reduce dedication. The mixed effects of the bright and dark sides are less considered, which needs to be integrated into the same theoretical model. Based on the TMGT effect of Pierce and Aguinis (2013), we explain the inverted U-shaped incentive of performance pressure on work vigor and dedication, further proving the TMGT effect in the performance pressure field.

In addition, we also found some intriguing results that performance pressure only had a significant negative correlation with vigor rather than the curvilinear relationship we assumed. One explanation is that the objects of our study are public sector employees with higher intrinsic motivation (Wright, 2007; Wright and Pandey, 2011; Wright et al., 2012). According to self determine theory, external performance pressure will have an erosive effect on intrinsic motivation and crowd out work enthusiasm (Deci and Ryan, 1985), so there is a negative correlation between the two variables. Another explanation is that based on the definition of two variables, dedication emphasizes the involvement and inspiring sense, while vigor refers to willingness, energy, and mental resilience during working (Schaufeli et al., 2006). That is to say, vigor is driven internally, it is the willingness and persistence to invest effort even in the face of difficulties; while dedication is driven externally, referring to being strongly involved in their works and experiencing a sense of significance, enthusiasm, inspiration, and challenge. Previous studies on job engagement tend to generalize the effects of performance pressure, ignoring the various effects on different dimensions of engagement. In contrast, our study provides a more nuanced explanation. In fact, the greater the performance pressure from the outside, the more suppressed the individual’s intrinsic motivation, which also leads to lower vigor; at the same time, because the performance pressure has a certain guiding effect on the individual’s achievement of goals, which makes the externally driven dedication present a priority. Therefore, externally driven dedication and dedication will show an inverted U-shaped relationship that increases first and then decreases.

Second, this paper reveals a new mediating mechanism (implicit incentives of mission valence) to cope with performance pressure. In the original context, the performance pressure theory is more applicable to the enterprise situation, but the research in the public sector is different. Because the public sector does not emphasize external material incentives, it pays more attention to internal spiritual incentives. Therefore, the internal mission valence of individuals has the function of buffering performance pressure. Existing studies focus more on the buffering effect of individual social resources, organizational support, personality traits, and other factors (Ness and Connelly, 2017; Mitchell et al., 2018). Employees with high emotional stability appraised performance pressure as a challenge rather than a threat, enhancing work engagement (Kundi et al., 2021). Others believe that approach motive (i.e., self-objectification) mediates the positive indirect effect of performance pressure on in-role behaviors; however, avoidance motive (i.e., workplace anxiety) mediates the negative indirect effect. Work meaningfulness strengthens the approach and avoidance tendencies that employees experience under underperformance pressure (Xu et al., 2021). Previous studies have provided comprehensive explanatory variables but do not consider mission value. As a unique source of stress for public sector employees, mission valence positively affects individual behaviors. Like public service motivation, it represents members’ expectations about the public sector’s goals, mission, vision, and values. Cultivating such mission valence can effectively cope with external pressure and improve dedication.

Third, we have a theoretical dialog with mission valence theory, which is consistent with Rainey and Steinbauer (1999), Wright and Pandey (2011), and Wright et al. (2012). Existing research on factors affecting mission valence is mostly analyzed from the perspective of transformational leadership (Wright et al., 2012; Pasha et al., 2017; Bosak et al., 2021). Transformational leadership plays a good role in mission valence, but not every public sector leader has a transformational leadership style. Suppose the research on how to shape MV only focuses on the impact of transformational leadership. In that case, the theoretical assumption is that the MV of public employees will be promoted in the hope of leaders. But in any organization, leaders are always fluid, and former and current leaders may have great differences in leadership, making it difficult to maintain the consistency of transformational leadership. Therefore, we believe only institutions can be sustainable and better shape mission valence. As a lasting system, performance appraisal plays a positive role in guiding mission valence. At the same time, our study also proves an inverted U-shaped relationship between performance pressure and mission valence. Moderate performance pressure is conducive to guiding the valence. In contrast, excessive pressure from performance management will crowd out mission valence and lead to short-sighted behavior - ignoring the organization’s mission and pursuing short-term performance goals.

### Practical implications

Our research findings have practical implications for public personnel management. Firstly, public personnel managers can make a stepped performance appraisal plan, and dynamically adjust the performance pressure with the improvement of the working ability of public sector employees. Managers should exert appropriate pressure in performance management, but prevent the influence of “too much of a good thing.” For example, if the content of the examination is too much, the frequency is too high, and the intensity is too large is not conducive to stimulating the work vitality. Therefore, managers should advocate for proportionality over extremity in the doctrine of the mean (Pierce and Aguinis, 2013) when conducting performance appraisal, and seek a balance of performance pressure. For example, performance management indicators should be adjusted according to the improvement of employees’ working ability, and a ladder-type assessment plan should be formed to control the pressure within a reasonable range and prevent excessive and insufficient situations. Use office automation software to dynamically grasp the completion of employees’ daily work tasks, reasonably design performance goals, and prevent the dark side of performance pressure.

Secondarily, managers should educate and train the organization’s mission to enhance the mission valence of public sector employees. Mission valence can be cultivated as an incentive resource. In personnel recruitment and selection, public human resource managers should select some public employees with high mission valence or public service motivation, ensuring the organization’s values coincide with those employees. Managers should redesign the hiring process to select public employees who spontaneously value the public sector’s mission. Besides, managers could increase the meaning or value employees perceive in their jobs through training and education. Moreover, it has been improved that transformational leadership can inspire miss valence (Wright et al., 2012; Pasha et al., 2017). It means that public sector leaders should pay attention to the cultivation of transformational leadership and be good at clearly communicating mission vision statements and ideal goals. Managers can incentivize public servants to work harder by highlighting how their works benefit society or how their performances contribute to the organization’s ability to operationalize values by job crafting (Robledo et al., 2019).

Thirdly, managers should pay attention to the guiding role of performance pressure in mission valence. This study found an inverted U-shaped mechanism between performance pressure and mission valence. When managers conduct performance management, they should pay attention to the crowding-out effect of performance pressure on mission valence. Managers should not exert excessive performance pressure, thus causing valence to be eroded by it; however, too little performance pressure will also affect mission valence. Therefore, managers must maintain a dynamic balance between “excess” and “deficiency.” Employees’ mental health will not get automatically improved *via* operational-level job stress interventions unless implemented measures correspond to the problems (e.g., work environmental challenges) they want to address (Akerstrom et al., 2021; Severin et al., 2021).

### Limitation and future research

Although our model explains the relationship between performance pressure, mission valence, vigor, and dedication, there is still room for improvement. On the one hand, since we used a cross-sectional design to collect data in this study, CMB may exist. Although we refer to the recommendation of Podsakoff et al. (2003) to use a program control to minimize the impact of CMB, the Harmon single factor test in the statistical control was also used to prove the influence of CMB is not significant, but it may still exist. Future studies can use different data sources to measure variables, such as performance pressure measured by superiors and mission valence evaluated by colleagues, to provide more accurate empirical evidence. On the other hand, future research can further explore the occurrence conditions and boundaries of the inverted U-shape mechanism. This paper reveals two sets of inverted U-shape relationships. However, when, where, and under what circumstances this mechanism occurs remains to be further examined. Future studies can further explore the moderating effects of variables such as career advancement, organizational support, and social support to clarify the boundary conditions of inverted U-shape more clearly.

## Conclusion

Performance pressure is an incentive tool that motivates employees in public sectors to engage in their work. Previous studies regard performance pressure as either a challenge that promotes functional behaviors or a threat that leads to dysfunctional behaviors. Our research made an integrated explanation to fill the gap, specifically concentrating on the curvilinear relationship between performance pressure and work vigor/dedication. Our findings reveal that performance pressure has an inverted U relationship with dedication and a negative effect on vigor. Besides, although performance pressure has a crow-out influence on vigor and dedication, we still find an important buffer mechanism – the intrinsic mission valence can mediate the inverted U relationship between performance pressure and dedication. Our research findings have practical implications for public personnel management. Public personnel managers, for example, can make a ladder-type performance appraisal plan for each public sector employee, dynamically adjust the performance pressure with the improvement of individual working ability, and control it within a reasonable range. At the same time, managers should carry out education and training about the mission of organizations, which strengthens the mission valence of public sector employees.

## Data availability statement

The original contributions presented in the study are included in the article/supplementary material, further inquiries can be directed to the corresponding author.

## Ethics statement

The studies involving human participants were reviewed and approved by the Academy of Neuroeconomics and Neuromanagement, Ningbo University. The patients/participants provided their written informed consent to participate in this study.

## Author contributions

ZS: conceptualization, data acquisition, data curation, analysis, writing-original draft preparation, and writing-revised draft preparation. BF: conceptualization, and data acquisition. All authors contributed to the article and approved the submitted version.

## Funding

This study was funded by grant 21&ZD089 from the major program of National Social Science Foundation of China.

## Conflict of interest

The authors declare that the research was conducted in the absence of any commercial or financial relationships that could be construed as a potential conflict of interest.

## Publisher’s note

All claims expressed in this article are solely those of the authors and do not necessarily represent those of their affiliated organizations, or those of the publisher, the editors and the reviewers. Any product that may be evaluated in this article, or claim that may be made by its manufacturer, is not guaranteed or endorsed by the publisher.

## Appendix A

Measurement items.

Vigor (VI).

(1) At my work, I feel bursting with energy. (VI1)

(2) At my job, I feel strong and vigorous. (VI2)

(3) When I get up in the morning, I feel like going to work. (VI3)

(4) I can continue working for very long periods at a time. (VI4)

(5) At my job, I am very resilient, mentally. (VI5)

(6) At my work, I always persevere, even when things do not go well. (VI6)

Dedication (DE).

(1) I find the work that I do full of meaning and purpose. (DE1)

(2) I am enthusiastic about my job. (DE2)

(3) My job inspires me. (DE3)

(4) I am proud of the work that I do. (DE4)

(5) To me, my job is challenging. (DE5)

Performance Pressure (PP).

(1) The pressures for performance in my workplace are high. (PP1)

(2) I feel tremendous pressure to produce results. (PP2)

(3) If I do not produce at high levels, my job will be at risk. (PP3)

(4) I would characterize my workplace as a results-driven environment. (PP4)

Mission Valence (MV).

(1) This organization provides valuable public service. (MV1)

(2) I believe that the priorities of this organization are quite important. (MV2)

(3) The work of this organization is not very significant in the broader scheme of things (MV3).

(4) For me, the mission of this organization is exciting. (MV4)
